# Variable Selection in Mixed‐Effects Location‐Scale and Location‐Shift Models

**DOI:** 10.1002/sim.70553

**Published:** 2026-04-28

**Authors:** Moritz Berger, Maria Iannario

**Affiliations:** ^1^ Core Facility Biostatistics, Central Institute of Mental Health, Medical Faculty Mannheim Heidelberg University Mannheim Germany; ^2^ Department of Political Sciences University of Naples Federico II Naples Italy

**Keywords:** clustered data, LASSO, location‐scale model, location‐shift model, proportional odds model, variable selection, variance heterogeneity

## Abstract

When ordinal responses to questionnaires structured on the basis of Likert scales show differing variability or heterogeneity in subgroups of the population, appropriate regression approaches that are able to take this issue into account are the location‐scale and location‐shift model. If data come in clusters, which causes within‐cluster variance, an additional cluster‐level random effect specification is due. Cumulative models for ordinal responses are considered assessing the responses in terms of mean level (or location), variability (or scale), heterogeneity (or dispersion) and in terms of random effects related to clusters. Furthermore, in order to reduce the complexity of the models, a variable selection procedure through adaptive fused LASSO‐type regularization is proposed. A case study with data from the Survey of Health, Ageing and Retirement in Europe is used to demonstrate the applicability of the models and the properties of the selection procedures. It is shown that variable selection by regularization produces stable parameter estimates and results that are easy to interpret in all model components. The performance of the proposed regularization approach is further assessed by means of a simulation study.

## 1 Introduction

Ordinal responses consist of values from a set of ordered categories, typically encoded as integer numbers, representing the evaluations of a group of respondents on a well‐defined list of items [1]. These data are commonly used to capture an individual's perceived level of perception, appreciation, or feeling. Ordinal scales are prevalent across various fields, among others, medicine and psychology, where questionnaires are often employed to assess services or opinions. The most popular tool for the analysis of ordinal responses is cumulative regression models [2]. In these models the response is derived from a coarser categorical version of an underlying latent continuous variable, which is linked to the covariates through a latent linear regression model. However, in several situations where the variability or the heterogeneity differs across subgroups of the population, which is also referred to as *varying dispersion*, classical cumulative models may show poor goodness‐of‐fit and inadequately represent the underlying probability structure. Two different classes of models have been proposed to take into account these issues: McCullagh's [2] proposal, the so‐called *location‐scale model*, extends the cumulative model by an additional scaling term in the denominator of the predictor function. It is also known as a heterogeneous choice model or heteroskedastic logit model [3, 4]. Alternatively, Tutz and Berger [5, 6] propose the so‐called *location‐shift model*, which accounts for varying dispersion by a shifting of thresholds. In their model weak dispersion can also be interpreted as a tendency toward the middle category, whereas strong dispersion means a tendency to the extreme categories. The two approaches represent different mechanisms for modeling heterogeneity in ordinal responses. In the location‐scale model, variability is handled through a subject‐specific scale parameter that inflates or shrinks the latent variability. In contrast, the location‐shift model keeps the latent scale fixed and represents changes in dispersion through systematic additive shifts of the thresholds, leading to an interpretation in terms of response patterns (middle vs. extreme category preference). For a broader conceptual framework and a detailed taxonomy of ordinal regression models that distinguishes these and related approaches, see Tutz [7]. Alternative approaches based on mixture models have also been introduced in the literature to account for decision uncertainty. Among these contributions, the approach proposed by Piccolo [8] and D'Elia and Piccolo [9] is particularly noteworthy, and has been subsequently extended in several directions, including hierarchical frameworks [10]. Here, we focus on both the location‐scale model and the location‐shift model and recent extensions to hierarchical structures, where data come in clusters of observation units [11, 12, 13]. We introduce a regularization approach based on adaptive LASSO‐type penalties that allows for variable selection in these hierarchical models. This is mainly motivated by the fact that already in settings with a moderate number of covariates the location‐scale and location‐shift models may result in a relatively large number of parameters compared to the sample size. As the models contain two different predictors, a location term and a dispersion term, it is crucial that the regularization approach treats these components in a flexible way and separate from each other.

The paper is organized as follows: Section 2 reviews the methodology in hierarchical settings starting from the random effects cumulative model. In Section 3 we introduce the regularization approach including LASSO and fused LASSO‐type penalty terms. To illustrate the alternative models and to give practitioners a step‐by‐step guidance on their application we present a case study using data from the Survey of Health, Ageing and Retirement in Europe [14] in Section 4. In Section 5 we show the results of a simulation study investigating variable selection properties. Finally, Section 6 concludes with a summary of the findings, implications for practice, and suggestions for future research.

## 2 Mixed Cumulative Regression

Let us in the following consider data that comes in clusters given by $\left(y_{\mathrm{ij}},x_{\mathrm{ij}}\right),i=1,\ldots ,n,j=1,\ldots ,N_{i}$, where $y_{\mathrm{ij}}\in \left\{1,k\right\}$ denotes the ordinal response of observation unit j in cluster i with k ordered categories. There are n different clusters with $N_{1},\ldots ,N_{n}$ observations. For example, in our case study presented in Section 4 the observations are grouped by country of the citizens. The vector of covariates $x_{\mathrm{ij}}$ may be constant or vary across measurements of one cluster. The most popular approach to relate the ordinal response to the covariates is the class of *cumulative regression models* introduced by McCullagh [2] in a pathbreaking paper on ordinal regression. For an overview on ordinal regression see also Agresti [1], Tutz [15] and Peyhardi et al. [16]. Including a cluster‐specific random intercept $b_{i}$ to account for unexplained (residual) heterogeneity between clusters, the random effects cumulative model [17] is given by

$$P\left(y_{\mathrm{ij}}\leq r|x_{\mathrm{ij}}\right)=F\left(\beta_{0r}-x_{\mathrm{ij}}^{⊤}\beta -b_{i}\right),\;r=1,\ldots ,k-1,$$

where F(⋅) is a distribution function, $\beta^{⊤}=\left(\beta_{1},\ldots ,\beta_{p}\right)$ is the vector of regression coefficients and $-\infty <\beta_{01}<\ldots <\beta_{0,k-1}<\infty$ are category‐specific intercepts that need to be strictly increasing. Tutz and Hennevogl [18] describe how the random effects cumulative model can be derived from an underlying latent regression model with continuous response variable. A widely applied choice for the distribution function is the logistic distribution function F(⋅)=exp(⋅)/(1+exp(⋅)), which yields the *proportional odds model* of the form

(1) $$\log\left(\frac{P\left(y_{\mathrm{ij}}\leq r|x_{\mathrm{ij}}\right)}{P\left(y_{\mathrm{ij}}>r|x_{\mathrm{ij}}\right)}\right)=\eta_{\mathrm{ijr}}=\beta_{0r}-x_{\mathrm{ij}}^{⊤}\beta -b_{i},\;r=1,\ldots ,k-1.$$

The representation in (1) uses the link function $g\left(P\left(y_{\mathrm{ij}}\leq r|x_{\mathrm{ij}}\right)\right)=\text{logit}\left(P\left(y_{\mathrm{ij}}\leq r|x_{\mathrm{ij}}\right)\right)$, which is given by $g\left(\cdot \right)=F^{-1}\left(\cdot \right)$. The use of the logistic distribution greatly simplifies the interpretation of the regression coefficients as well as has interesting properties with regard to robustness [19, 20]. If $\beta_{s}>0$, increasing $x_{\mathrm{ijs}}$ by one unit (all other model components fixed) implies that the cumulative odds $P\left(y_{\mathrm{ij}}\leq r|x_{\mathrm{ij}}\right)/P\left(y_{\mathrm{ij}}>r|x_{\mathrm{ij}}\right)$ decrease by the factor $\exp\left(\beta_{s}\right)$, which means that the probability for higher categories increases. This effect is the same across all categories: a positive $\beta_{s}$ implies that an increase in $x_{\mathrm{ijs}}$ uniformly shifts the probability mass toward higher response categories, regardless of the specific threshold r. If $\beta_{s}<0$, all cumulative odds increase by the factor $\exp\left(\beta_{s}\right)$, implying a shift of the probability mass to lower response categories. The random intercepts in (1) are assumed to be normally distributed with zero mean and variance $\sigma^{2}$, $b_{i}∼N\left(0,\sigma^{2}\right)$. Presence of the random intercepts means that the category‐specific thresholds are simultaneously shifted yielding the thresholds $\beta_{01}-b_{i},\ldots ,\beta_{0,k-1}-b_{i}$. Thus the resulting category‐specific intercepts vary across clusters. The proportional odds model with cluster‐specific random intercept has been studied previously, among others, by Jansen [21], Ezzet and Whitehead [22], and Agresti and Lang [23]. In recent years, the model has also been used for numerous applications in many research areas, see, for example, Doussau et al. [24], Augustin et al. [25], and Zewude and Debusho [26]. A comparison of available statistical software packages for proportional odds model with random effects has been provided by Li et al. [27]. Furthermore, Tutz and Hennevogl [18] considered an extension of the model by random slopes. In the following, we will focus on cumulative models with logistic distribution function and cluster‐specific random intercepts.

### 2.1 Random Effects Location‐Scale Model

Although the proportional odds model is conceptually very simple, it often shows a poor goodness‐of‐fit and does not satisfactorily represent the underlying probability structure. A lack of fit can be caused by differing variability (also referred to as varying dispersion) in subgroups of the population. This form of misspecification can be avoided by explicit modeling of dispersion effects. A model that accounts for varying dispersion is the location‐scale model [2, 28]. Built upon this model, Hedeker et al. [11, 29] proposed a random effects location‐scale model for clustered data. The variant of the model with cluster‐specific random intercepts, which we are considering here, has the form

(2) $$\log\left(\frac{P\left(y_{\mathrm{ij}}\leq r|x_{\mathrm{ij}},z_{\mathrm{ij}}\right)}{P\left(y_{\mathrm{ij}}>r|x_{\mathrm{ij}},z_{\mathrm{ij}}\right)}\right)=\eta_{\mathrm{ijr}}=\frac{\beta_{0r}-x_{\mathrm{ij}}^{⊤}\beta -b_{i,\ell }}{\exp\left(z_{\mathrm{ij}}^{⊤}\alpha +b_{i,d}\right)},\;r=1,\ldots ,k-1,$$

where $z_{\mathrm{ij}}$ is an additional vector of covariates. The model in (2) contains the familiar location term in the numerator and an additional scaling term $\tau_{\mathrm{ij}}=\exp\left(z_{\mathrm{ij}}^{⊤}\alpha +b_{i,d}\right)$ in the denominator of the predictor function, which determines the variance of the probability distribution as a linear function of the covariates $z_{\mathrm{ij}}$. The random effects location‐scale model includes two random intercepts, $b_{i}^{⊤}=\left(b_{i,\ell },b_{i,d}\right)∼N\left(0,\sum_{b}\right)$, one in the location term and one in the dispersion term with zero mean and variance–covariance matrix $\sum_{b}=\left(\begin{array}{cc} \sigma_{\ell }^{2} & \sigma_{\ell ,d}\\ \sigma_{\ell ,d} & \sigma_{d}^{2} \end{array}\right)$. As in the simple model, the random intercept in the location term represents between‐cluster variability with a shifting of the thresholds, while the dispersion term $\tau_{\mathrm{ij}}$ represents within‐cluster variability. Presence of the random intercepts in the dispersion term means that the within‐cluster variance is allowed to differ across clusters [11]:

- If $b_{i,\ell }\to -\infty$ one obtains (all other model components fixed) the probabilities $P\left(y_{\mathrm{ij}}=1|x_{\mathrm{ij}},z_{\mathrm{ij}}\right)=1$, and $P\left(y_{\mathrm{ij}}=2|x_{\mathrm{ij}},z_{\mathrm{ij}}\right)=\ldots =P\left(y_{\mathrm{ij}}=k|x_{\mathrm{ij}},z_{\mathrm{ij}}\right)=0$. In contrast, if $b_{i,\ell }\to \infty$ one obtains (all other model components fixed) the probabilities $P\left(y_{\mathrm{ij}}=k|x_{\mathrm{ij}},z_{\mathrm{ij}}\right)=1$, and $P\left(y_{\mathrm{ij}}=1|x_{\mathrm{ij}},z_{\mathrm{ij}}\right)=\ldots =P\left(y_{\mathrm{ij}}=k-1|x_{\mathrm{ij}},z_{\mathrm{ij}}\right)=0$. Thus, the size of $b_{i,\ell }$ indicates the cluster‐specific preference for high categories.
- If $b_{i,d}\to -\infty$ one obtains (all other model components fixed) the probabilities $P\left(y_{\mathrm{ij}}=k/2|x_{\mathrm{ij}},z_{\mathrm{ij}}\right)=P\left(y_{\mathrm{ij}}=k/2+1|x_{\mathrm{ij}},z_{\mathrm{ij}}\right)=0.5$ (for k even) and $P\left(y_{\mathrm{ij}}=m|x_{\mathrm{ij}},z_{\mathrm{ij}}\right)=1$, with m=⌊k/2⌋+1 denoting the middle (for k odd). If $b_{i,d}\to \infty$ one obtains (all other model components fixed) $P\left(y_{\mathrm{ij}}=1|x_{\mathrm{ij}},z_{\mathrm{ij}}\right)=P\left(y_{\mathrm{ij}}=k|x_{\mathrm{ij}},z_{\mathrm{ij}}\right)=0.5$, Thus, the size of $b_{i,d}$ determines the cluster‐specific variability with the responses tending to the middle or the extreme categories.

Regarding the regression coefficients, it is important to note that, if $x_{\mathrm{ij}}$ and $z_{\mathrm{ij}}$ are distinct, the interpretation of the location effects β in terms of cumulative odds remains the same as in the simple model (1). The scaling term in (2) determines within‐cluster variance. If $\alpha_{s}>0$, increasing $z_{\mathrm{ijs}}$ by one unit (all other model components fixed) implies increased variability, and vice versa. Although the scaling term is typically motivated from variance heterogeneity it can also be seen as representing interactions or effect modification [30, 31].

### 2.2 Random Effects Location‐Shift Model

As an alternative to account for varying dispersion, Tutz and Berger [6, 32] proposed the location‐shift model. Including cluster‐specific random intercepts to account for unexplained (residual) heterogeneity, which we are considering here, yields the location‐shift model with predictor

(3) $$\eta_{\mathrm{ijr}}=\beta_{0r}-x_{\mathrm{ij}}^{⊤}\beta -b_{i,\ell }+\left(r-k/2\right)\;\left(z_{\mathrm{ij}}^{⊤}\alpha +b_{i,d}\right),\;r=1,\ldots ,k-1.$$

The model in (3) contains the familiar location term with random intercept $b_{i,\ell }$, which reflects the tendency to low or high categories, and a scaled shifting term depending on r with random intercept $b_{i,d}$, which modifies the thresholds and reflects the tendency to the middle or extreme categories. The random intercepts $b_{i,d}$ explicitly refer to the shifting of thresholds on the cluster level, while the linear term $z_{\mathrm{ij}}^{⊤}\alpha$ determines the shifting on an individual level. As before, we assume that $b_{i}^{⊤}=\left(b_{i,\ell },b_{i,d}\right)∼N\left(0,\sum_{b}\right)$. For illustration, let us consider the simple case k=4 with k/2=2, for which the modified thresholds have the form

$$\begin{array}{ll} & \beta_{01}-z_{\mathrm{ij}}^{⊤}\alpha -b_{i,d},\\ & \beta_{02},\\ & \beta_{03}+z_{\mathrm{ij}}^{⊤}\alpha +b_{i,d}. \end{array}$$

In this example the middle threshold $\beta_{02}$ remains fixed, but the lower and upper threshold are shifted by $\delta_{\mathrm{ij}}=z_{\mathrm{ij}}^{⊤}\alpha +b_{i,d}$. The model postulates the ordering of thresholds such that $-\infty <\beta_{01}-\delta_{\mathrm{ij}}<\beta_{02}<\beta_{03}+\delta_{\mathrm{ij}}<\infty$. If $\delta_{\mathrm{ij}}>0$ the intervals defined by the thresholds are widened, indicating weaker dispersion and therefore more concentration in the middle. On the other hand, if $\delta_{\mathrm{ij}}<0$ the intervals are shrunk, indicating stronger dispersion and therefore more concentration in the extreme categories. Regarding the random intercept, if $b_{i,d}\to \infty$ (all other model components fixed) one obtains $P\left(y_{\mathrm{ij}}=2|x_{\mathrm{ij}},z_{\mathrm{ij}}\right)=P\left(y_{\mathrm{ij}}=3|x_{\mathrm{ij}},z_{\mathrm{ij}}\right)=0.5$ with the whole probability mass in the middle categories. If $b_{i,d}\to -\infty$ (all other model components fixed) one obtains $P\left(y_{\mathrm{ij}}=1|x_{\mathrm{ij}},z_{\mathrm{ij}}\right)=P\left(y_{\mathrm{ij}}=4|x_{\mathrm{ij}},z_{\mathrm{ij}}\right)=0.5$ with the whole probability mass in the extreme categories. The location‐shift model (3) constitutes a variant of the class of cumulative models accounting for response styles, which was introduced by Schauberger and Tutz [13] for the analysis of repeated item response data. Furthermore, the class of location‐shift models represents an interesting intermediate alternative between the proportional odds and non‐proportional odds model. Details on this are outlined in Tutz and Berger [32].

## 3 Regularized Estimation

In both models, the location‐scale model (2) and the location‐shift model (3) there are two different sets of covariates, the vector $x_{\mathrm{ij}}$ determining the location and the vector $z_{\mathrm{ij}}$ determining the dispersion. In applications, however, there is only one set of covariates and it is typically not known which variables have an impact on which of the two components. Moreover, specifying a predictor for the location term and for the dispersion term may result in large number of parameters relative to the sample size even in settings with a moderate number of covariates. This calls for introducing a specific variable selection mechanism in the estimation procedure, which is the main methodological contribution in the present paper.

### 3.1 Penalized Marginal Likelihood

For the clustered data $\left(y_{\mathrm{ij}},x_{\mathrm{ij}}\right),i=1,\ldots ,n,j=1,\ldots ,N_{i},$ and based on the assumption of conditional independence given the random effects, the marginal likelihood for one cluster (in our case study for one country), using either of the two models, can be expressed as an integral over the likelihood of the form

$$L_{i}\left(\beta ,\alpha ,\sum_{b}\right)=\int \int \prod_{j=1}^{N_{i}}\prod_{r=1}^{k}P{\left(y_{\mathrm{ij}}=r|\beta ,\alpha ,\sum_{b}\right)}^{\Delta_{\mathrm{ijr}}}\;f\left(b_{i,\ell },b_{i,d}\right)\;{\mathrm{db}}_{i,\ell }\;{\mathrm{db}}_{i,d},$$

where $\Delta_{\mathrm{ijr}}=1$ if $y_{\mathrm{ij}}=r$ and $y_{\mathrm{ijr}}=0$, otherwise [15, 33]. The term $f\left(b_{i,\ell },b_{i,d}\right)$ denotes the two‐dimensional probability density function of the random intercepts, $N\left(0,\sum_{b}\right)$. The probabilities for each response category are derived by the differences $P\left(y_{\mathrm{ij}}=r|\beta ,\alpha ,\sum_{b}\right)=P\left(y_{\mathrm{ij}}\leq r|\beta ,\alpha ,\sum_{b}\right)-P\left(y_{\mathrm{ij}}\leq r-1|\beta ,\alpha ,\sum_{b}\right),r=1,\ldots ,k$. Note that by design $P\left(y_{\mathrm{ij}}\leq k|\beta ,\alpha ,\sum_{b}\right)=1$ and is not explicitly modeled. Then the total log‐likelihood becomes

(4) $$\ell \left(\beta ,\alpha ,\sum_{b}\right)=\sum_{i=1}^{n}\log\left(L_{i}\left(\beta ,\alpha ,\sum_{b}\right)\right).$$

To obtain a sparse representation and in particular variable selection with regard to the location and the dispersion term, we do not consider the raw log‐likelihood (4) but a penalized log‐likelihood of the form

(5) $$\ell_{p}\left(\beta ,\alpha ,\sum_{b}\right)=\ell \left(\beta ,\alpha ,\sum_{b}\right)-J_{\lambda_{\ell },\lambda_{d}}\left(\alpha ,\beta \right),$$

where $J_{\lambda_{\ell },\lambda_{d}}\left(\alpha ,\beta \right)$ represents a penalty term that depends on the scalar tuning parameters $\lambda_{\ell }$ and $\lambda_{d}$. The specific form of the penalty term determines the property of the penalized estimator. To allow for a different degree of regularization in the two model components, we propose to use two separate LASSO‐type penalties on the location parameters β and the dispersion parameters α. This includes all continuous covariates and dummy variables of binary covariates. In addition, for all ordinal covariates (let's say there are q) we specify a fusion penalty that enforces the building of clusters of categories that share the same effect [34, 35, 36]. This results to an adaptive penalty of the form

(6) $$\begin{array}{ll} J_{\lambda_{\ell },\lambda_{d}}\left(\alpha ,\beta \right) & =\lambda_{\ell }\sum_{r=1}^{p}w_{r}^{\left(l\right)}\;|\beta_{r}|+\lambda_{\ell }\sum_{s=1}^{q}\sum_{u<v}w_{\mathrm{suv}}^{\left(l\right)}\;|\beta_{\mathrm{su}}-\beta_{\mathrm{sv}}|\\ & \;+\lambda_{d}\sum_{r=1}^{p}w_{r}^{\left(d\right)}\;|\alpha_{r}|+\lambda_{d}\sum_{s=1}^{q}\sum_{u<v}w_{\mathrm{suv}}^{\left(d\right)}\;|\alpha_{\mathrm{su}}-\alpha_{\mathrm{sv}}|, \end{array}$$

where the elements $w_{r}^{\left(l\right)}=1/|{\hat{\beta }}_{r}|$, $w_{\mathrm{suv}}^{\left(l\right)}=1/|{\hat{\beta }}_{\mathrm{su}}-{\hat{\beta }}_{\mathrm{sv}}|$, $w_{r}^{\left(d\right)}=1/|{\hat{\alpha }}_{r}|$ and $w_{\mathrm{suv}}^{\left(d\right)}=1/|{\hat{\alpha }}_{\mathrm{su}}-{\hat{\alpha }}_{\mathrm{sv}}|$ are adaptive weights determined by the inverse of the unpenalized maximum likelihood estimates. The strength of the penalty terms is determined by the size of the tuning parameters $\lambda_{\ell }$ and $\lambda_{d}$ and varies with the weights for different coefficients. The idea behind the weights is that parameters with estimates converging to zero yield strong penalization, while relevant effects are penalized less severely. Without a penalty (i.e., $\lambda_{\ell }=\lambda_{d}=0$) no variable selection is enforced. The optimal tuning parameters can be chosen by subsampling or cross‐validation using the predictive log‐likelihood as the criterion to be evaluated. Zou [37] proved that the LASSO is consistent in terms of variable selection when used with adaptive weights. In Tutz et al. [38], the improved performance of adaptive penalties was empirically confirmed for the multinomial logit model.

### 3.2 Computation of ML Estimates and Inference

To maximize the penalized marginal log‐likelihood (5), integration over the random‐effects distribution must be performed. As we assume normally distributed random intercepts, Gauß‐Hermite quadrature can be applied to approximate the integral to a specific degree of accuracy. A quadrature method approximates an integral by the weighted sum over an adequate number of points on the abscissas for the random effects. In our implementation, we make use of the SAS PROC NLMIXED, which offers a general framework for fitting nonlinear mixed effects models. For computing the integral PROC NLMIXED uses adaptive Gaussian quadrature, which centers the integral at the empirical Bayes estimate of $b_{i}$ [39]. We set the number of quadrature points equal to one. For optimization PROC NLMIXED performs a quasi‐newton algorithm, which also involves computating the first‐order derivatives of the quadrature approximation. To conduct inference on the regression coefficients, PROC NLMIXED computes approximate standard errors using the delta method and provides p‐values and confidence limits based on the corresponding t‐statistics. In our specification of PROC NLMIXED, provided in Appendix A to this article, the user can assign the tuning parameters $\lambda_{\ell }$ and $\lambda_{d}$, the number of maximal function calls (maxiter, maxfunc), and starting values for all model parameters.

## 4 Case Study

**FIGURE 1:**
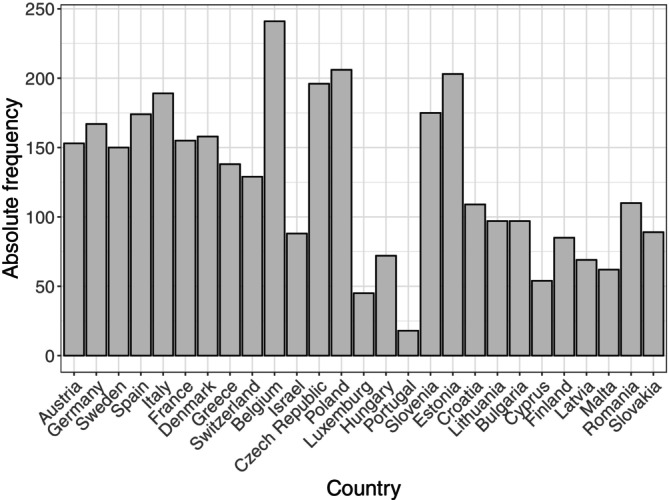
Analysis of SHARE. Absolute frequencies of individuals per country included in the analysis data set with n=3429 observations. The data contains between *n* = 18 (Portugal) and *n* = 241 (Belgium) individuals per country.

The data considered here come from the seventh wave of SHARE (Survey of Health, Ageing and Retirement in Europe^1^), collected in 2017 [14, 40]. SHARE is a panel survey collecting detailed cross‐national information on the health, socio‐economic status and family networks of people aged 50 and over from a large group of European countries. The main object is to investigate the impact of these factors on the aging process and to explore differences between European countries in dealing with the consequences of population aging. As reported in several studies (see among others [41]), a wide range of explanatory variables was collected in the survey. In addition to standard demographic information and socio‐economic status (among others, employment status, household income, and properties), various health‐related aspects (among others, chronic diseases, limitations in activity, and cognitive abilities) were gathered. We analyze a sample consisting of n=3429 respondents living in 27 countries. It is characterized by 56.6% of female respondents, with an average age of 68.1 years (SD = 9.7 years). Figure 1 shows the absolute frequency of observations by country. The analyzed data set contains between n=18 (Portugal) and n=241 (Belgium) individuals per country.

The focus of the present analysis will be on modeling the perception of one's health status as the outcome variable of interest, which is measured on an ordinal scale from 1 (excellent) to 5 (poor). We include the following eight covariates: age (in years), sex (0: male, 1: female), body mass index (BMI), years of education, ADL index (number of limitations with activities of daily living), IADL index (number of limitations in instrumental activities of everyday life), life satisfaction, and hand grip (which is a measure of physical health). While age, BMI, years of education, ADL index, IADL index, and hand grip were measured on a continuous scale, life satisfaction was measured on a scale from 1 (completely dissatisfied) to 7 (completely satisfied) and treated as an ordinal variable. Summary statistics of the outcome variable and covariates used in the analysis are presented in Table 1.

**TABLE 1:** Analysis of SHARE. Description and summary statistics of the outcome variable and the eight covariates.

Variable	Summary statistics
Health status	
1:	182 (5.3%)
2:	621 (18.1%)
3:	1275 (37.2%)
4:	1001 (29.2%)
5:	350 (10.2%)

	Min	Q1	Median	Q3	Max	Mean	SD
Age (years)	38	61	67	75	100	68.1	9.7
BMI (kg/m^2^)	15	24	27	30	51	27.3	4.7
Education (years)	0	8	11	14	25	11.1	4.2
ADL index	0	0	0	0	6	0.2	0.7
IADL index	0	0	0	0	9	0.4	1.1
Hand grip	1	25	31	40	85	32.7	11.6

Sex	
Male	1487 (43.4%)
Female	1942 (56.6%)
Life satisfaction	
1:	150 (4.4%)
2:	297 (8.7%)
3:	249 (7.3%)
4:	596 (17.4%)
5:	1071 (31.2%)
6:	542 (15.8%)
7:	524 (15.3%)

*Note:* The sample consists of n=3429 respondents living in 27 countries (Q1 = first quartile, Q3 = third quartile).

**FIGURE 2:**
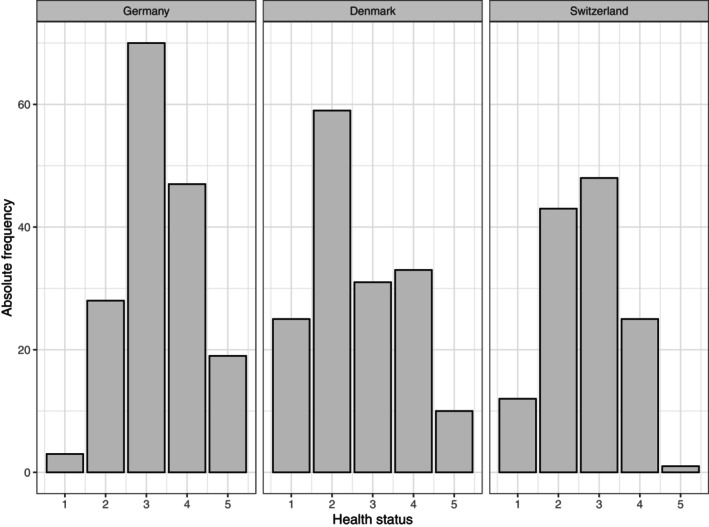
Analysis of SHARE. Probability distribution of the outcome variable (perception of one's health status) in three different countries. The normalized Gini heterogeneity index results in 0.88 (Germany), 0.88 (Switzerland), and 0.94 (Denmark).

To illustrate the problem of varying dispersion, we consider the probability distribution of the outcome variables in three exemplary countries (Germany, Denmark, and Switzerland). It is seen that there is a stronger preference for high categories (poorer health) in Germany compared to Denmark and Switzerland. In Denmark and Switzerland, the average tendency is quite similar, whereas there is a tendency to the extreme categories in Denmark compared to Switzerland and Germany. This is reflected in the normalized Gini heterogeneity index [42], which is 0.88 (Germany), 0.88 (Switzerland), and 0.94 (Denmark), which indicates stronger dispersion in Denmark (Figure 2).

For our evaluations we split the analysis data set randomly into a “training,” a “validation,” and a “test” sample with equal size of $\tilde{n}=1143$, each. We draw the samples on an individual level ensuring that each country is represented in all the three sets. The training sample was used for fitting and model comparison, the validation sample was used to determine the optimal tuning parameters, and the test sample was subsequently used to evaluate predictive performance and to derive confidence intervals. In two sensitivity analyses we generated the training sample and validation sample as before with equal size of $\tilde{n}=1393$, but left out all individuals of three countries (Belgium, Czech Republic, Poland; $\tilde{n}=643$) for testing (sensitivity analysis A), and all individuals of four countries (Italy, Slovenia, Sweden, Switzerland; $\tilde{n}=643$) for testing (sensitivity analysis B), see Section 4.3.

### 4.1 Model and Variable Selection

In the first step of our analysis we considered the training sample and compared different models without variable selection. Specifically, we fitted the simple proportional odds model, the proportional odds model with country‐specific random intercept (1), the location‐scale model with country‐specific random intercept (2), and the location‐shift model with country‐specific random intercept (3). In case of the models with dispersion, all eight covariates were included in the location term as well as in the scaling term and the shifting term, respectively. This results in 17 parameters (four intercept coefficients and 13 regression coefficients) to be estimated in the simple proportional odds model, 18 parameters in the model with random intercept, and 33 parameters (four intercepts, 13 location effects, 13 dispersion effects, and three variance–covariance parameters) in the models with dispersion.

A comparison of the goodness‐of‐fit measures reported in Table 2 shows that country‐specific effects should not be neglected, as the deviance (minus two times the log‐likelihood), AIC and BIC of the models with random intercepts were considerably lower. In the further steps of our analysis we therefore omitted models without random effects. In addition, the location‐scale and the location‐shift model exhibited a much better fit than the proportional odds model. As the difference in AIC is >10 (location‐scale: 24 and location‐shift: 23), there is essentially no empirical support for the model without dispersion [43].

**TABLE 2:** Analysis of SHARE. Goodness‐of‐fit of the cumulative models without variable selection evaluated on the training sample with $\tilde{n}=1143$ observations.

Model	Deviance	AIC	BIC
Proportional odds model	2862	2895	2981
Random effects proportional odds model	2815	2882	2924
Location‐scale model with random intercept	2800	2866	2909
Location‐shift model with random intercept	2795	2861	2904

*Note:* All models included the covariates age, sex, BMI, years of education, ADL index, IADL index, hand grip, and life satisfaction. In case of the models with dispersion all eight covariates were included in the location term as well as in the scaling term and the shifting term, respectively. This results in 17 parameters to be estimated in the simple proportional odds model, 18 parameters in the model with random intercept, and 33 parameters in the models with dispersion.

In the second step of our analysis we considered the three models with country‐specific intercepts and introduced the proposed variable selection mechanism. For this we applied the penalized likelihood with adaptive $L_{1}$ penalty for the continuous covariates (and sex) and the adaptive fusion penalty for the ordinal covariate life satisfaction. Before fitting the models as described in Section 3.2 we standardized all continuous covariates and dummy variables referring to sex and life satisfaction to have mean 0 and variance 1. The penalty parameters were optimized on a two‐dimensional grid with values, $\lambda_{l},\lambda_{d}\in \left\{-10,-9,\ldots ,-2,-1\right\}$ (on the logarithmic scale), calculating the predictive log‐likelihood values on the validation sample.

**FIGURE 3:**
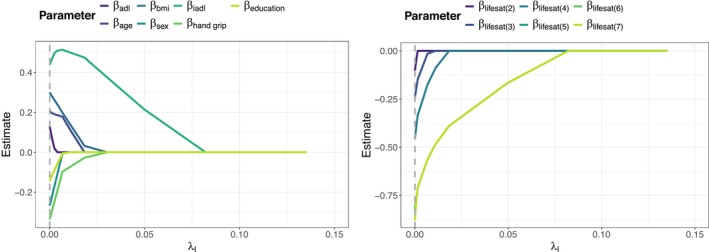
Analysis of SHARE. Coefficient paths of the location parameters for the continuous covariates and sex (left), and life satisfaction (right) when fitting the penalized proportional odds model with country‐specific random intercept to the learning sample. The optimal penalty parameter ($\lambda_{l}=\exp\left(-10\right)$) is marked by the dashed vertical line.

**FIGURE 4:**
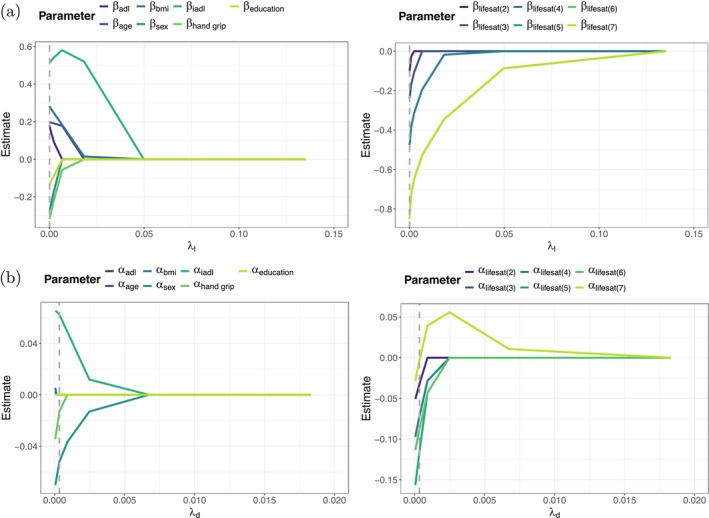
Analysis of SHARE. Coefficient paths of the location parameters (a) and the dispersion parameters (b) for the continuous covariates and sex (left), and life satisfaction (right) when fitting the penalized location‐scale model with country‐specific random intercept to the learning sample. The optimal penalty parameter is marked by the dashed vertical line, respectively. In (a) the penalty parameter in the dispersion term was set to the optimal value of $\lambda_{d}=\exp\left(-8\right)$, in (b) the penalty parameter in the location term was set to the optimal value of $\lambda_{\ell }=\exp\left(-9\right)$. Note that for better readability the *x*‐axis was truncated on the right.

**FIGURE 5:**
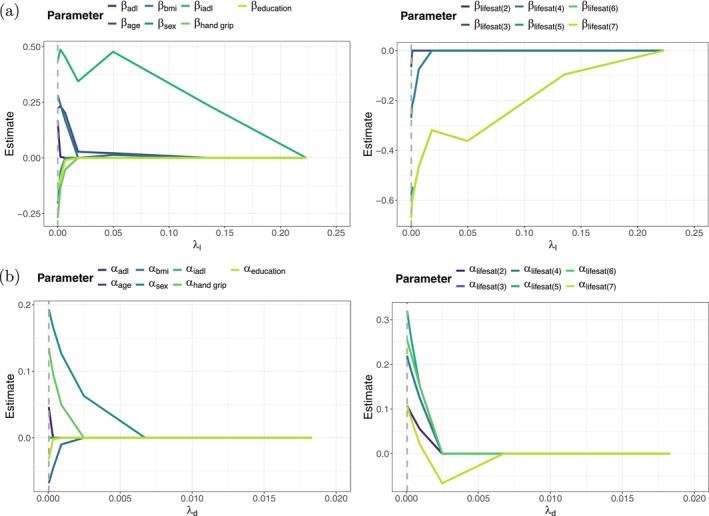
Analysis of SHARE. Coefficient paths of the location parameters (a) and the dispersion parameters (b) for the continuous covariates and sex (left), and life satisfaction (right) when fitting the penalized location‐shift model with country‐specific random intercept to the learning sample. The optimal penalty parameter is marked by the dashed vertical line, respectively. In (a) the penalty parameter in the dispersion term was set to the optimal value of $\lambda_{d}=\exp\left(-10\right)$, in (b) the penalty parameter in the location term was set to the optimal value of $\lambda_{\ell }=\exp\left(-9\right)$. Note that for better readability the *x*‐axis was truncated on the right.

Figure 3 shows the coefficient paths of the location parameters β when fitting the penalized proportional odds model with country‐specific random intercepts to the training sample. According to the optimal penalty parameter ($\lambda_{l}=\exp\left(-10\right)$) the optimal model is the model with minimal penalization, where none of the covariates is excluded from the predictor and only the levels 5 and 6 of life satisfaction were collapsed to one category; these adjacent categories exhibited very similar response frequencies and indistinguishable empirical behavior. Collapsing them helps stabilize the estimation without compromising the substantive interpretation of the scale. Figures 4 and 5 show the coefficient paths of the location parameters β (panels (a)) and the dispersion parameters α (panels (b)) when fitting the penalized location‐scale model and the penalized location‐shift model with country‐specific random intercept. It is again seen that in both cases the evaluation on the validation sample indicates only little penalization in the location term. In the location‐shift model only the second level of life satisfaction was excluded; in the location‐scale model (as in the model without dispersion) the levels 5 and 6 of life satisfaction were collapsed to one category. Regarding the dispersion term, however, the picture changes. The optimal penalty parameters in the dispersion term were estimated to be $\lambda_{d}=\exp\left(-8\right)$ (location‐scale) and $\lambda_{d}=\exp\left(-10\right)$ (location‐shift), which causes a selection of covariates as well as a fusion of effects in life satisfaction. In the location‐scale model (Figure 4b) age, BMI, years of education and ADL index were excluded from the model; furthermore levels 3 and 4 of life satisfaction were collapsed to one category having the same dispersion effect on the outcome variable. In the location‐shift model (Figure 5b) only age was excluded from the model. Importantly, both models reveal weaker dispersion and therefore a tendency to the middle categories for individuals with a higher life satisfaction compared to individuals that are completely dissatisfied (Life satisfaction = 1), as the estimates of the dispersion effects α are negative in the location‐scale model and positive in the location‐shift model.

To evaluate the performance of the three penalized cumulative models, we calculated the predictive deviance (on the population level) given by

$$\text{Deviance}=-2\;\sum_{i=1}^{n}\sum_{j=1}^{N_{i}}\log\left(\prod_{r=1}^{k}P{\left(y_{\mathrm{ij}}=r|\hat{\beta },\hat{\alpha },{\hat{\sum }}_{b}\right)}^{\Delta_{\mathrm{ijr}}}\right),$$

and the average ranked probability score [44] quantifying the agreement between the fitted probability distribution and the observations, which is defined by

$$\mathrm{RPS}=\frac{1}{n\;N_{i}}\sum_{i=1}^{n}\sum_{j=1}^{N_{i}}\left(\sum_{r=1}^{k}{\left(P\left(y_{\mathrm{ij}}\leq r|\hat{\beta },\hat{\alpha },{\hat{\sum }}_{b}\right)-I\left(y_{i}\leq r\right)\right)}^{2}\right),$$

on the test sample. The RPS takes the whole predictive distribution (like sharpness and calibration) into account. Lower values of the RPS indicate better predictive performance. The results are given in Table 3. While there were hardly any differences in the RPS (the values only differ on the third or fourth decimal place), the deviance was lowest for the location‐scale model with a difference of 9.84 to the model without dispersion and 4.94 to the location‐shift model.

**TABLE 3:** Analysis of SHARE. Predictive performance of the penalized cumulative models evaluated on the test sample with $\tilde{n}=1143$ observations.

Model	Deviance	RPS
Random effects proportional odds model	2956.13	0.47735
Location‐scale model with random intercept	2946.29	0.47570
Location‐shift model with random intercept	2951.23	0.47566

*Note:* The penalized location‐scale model excluded the covariates age, BMI, years of education, and ADL index from the scaling term resulting in 27 parameters, the penalized location‐shift model only excluded age from the shifting term resulting in 31 parameters. Lower values of deviance and RPS indicate better predictive accuracy.

### 4.2 Fit of the Selected Models

**FIGURE 6:**
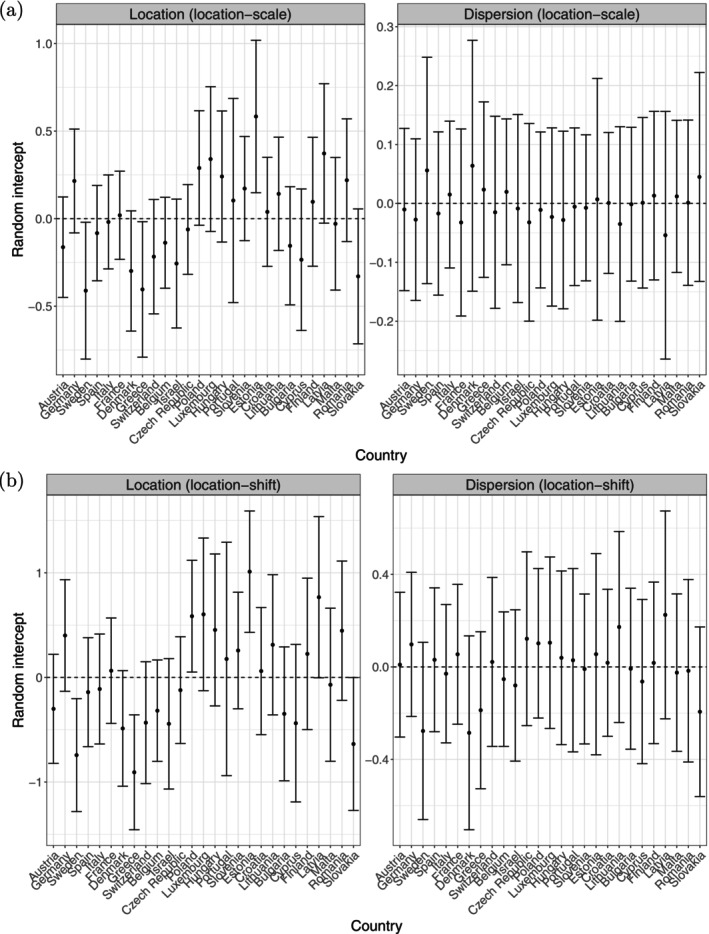
Analysis of SHARE. Estimated random country‐specific intercepts with 95% confidence intervals (CI) of the final location‐scale model (a) and the final location‐shift model (b).

In the final step of our analysis, we fitted both the final location‐scale model and location‐shift model to the original training sample (with covariates in their original, non‐standardized form) in order to obtain coefficient estimates and to the test sample to derive standard errors for the construction of confidence intervals. For this we refitted the selected models by PROC NLMIXED without penalization, yielding standard errors for the regression coefficients and standard errors for the random effect estimates (empirical Bayes estimates). The results are reported in Tables 4 and 5 and Figure 6.

**TABLE 4:** Analysis of SHARE. Coefficient estimates, exponential coefficients, and 95% confidence intervals (CI) of the final location‐scale model with country‐specific intercepts.

Location effects	Covariate	$\hat{\beta }$	$\exp\left(\hat{\beta }\right)$	95% CI
	Age	0.0111	1.0112	[0.9984; 1.0241]
	BMI	0.0312	1.0317	[1.0106; 1.0532]
	Education	−0.0193	0.9809	[0.9621; 1.0001]
	ADL index	0.1390	1.1491	[0.9599; 1.3757]
	IADL index	0.2493	1.2831	[1.1511; 1.4302]
	Hand grip	−0.0142	0.9859	[0.9738; 0.9980]
	Sex (female)	−0.2848	0.7521	[0.5932; 0.9536]
	Life satisfaction (2)	−0.1985	0.8200	[0.5242; 1.2826]
	Life satisfaction (3)	−0.4893	0.6131	[0.3708; 1.0135]
	Life satisfaction (4)	−0.6699	0.5118	[0.3117; 0.8402]
	Life satisfaction (5, 6)	−0.9936	0.3702	[0.1934; 0.7087]
	Life satisfaction (7)	−1.2406	0.2892	[0.1417; 0.5904]

Dispersion effects	Covariate	$\hat{\alpha }$	$\exp\left(\hat{\alpha }\right)$	95% CI
	IADL index	0.0588	1.0606	[0.9889; 1.1373]
	Hand grip	−0.0039	0.9961	[0.9882; 1.0041]
	Sex (female)	−0.1657	0.8473	[0.7144; 1.0049]
	Life satisfaction (2)	−0.2021	0.8170	[0.5603; 1.1914]
	Life satisfaction (3, 4)	−0.3220	0.7247	[0.5181; 1.0136]
	Life satisfaction (5)	−0.3678	0.6922	[0.4947; 0.9687]
	Life satisfaction (6)	−0.3193	0.7267	[0.5109; 1.0335]
	Life satisfaction (7)	−0.1100	0.8959	[0.6292; 1.2755]

*Note:* The model excluded the covariates age, BMI, years of education, and ADL index from the scaling term, resulting in 27 parameters. Parameter estimates were obtained from the training sample; the corresponding standard errors were obtained from the test sample.

**TABLE 5:** Analysis of SHARE. Coefficient estimates, exponential coefficients, and 95% confidence intervals (CI) of the final location‐shift model with country‐specific intercepts.

Location effects	Covariate	$\hat{\beta }$	$\exp\left(\hat{\beta }\right)$	95% CI
	Age	0.0231	1.0234	[1.0088; 1.0382]
	BMI	0.0605	1.0623	[1.0349; 1.0905]
	Education	−0.0345	0.9661	[0.9377; 0.9954]
	ADL index	0.2527	1.2876	[0.7726; 2.1458]
	IADL index	0.4092	1.5056	[1.2004; 1.8884]
	Hand grip	−0.0233	0.9770	[0.9610; 0.9933]
	Sex (female)	−0.4154	0.6601	[0.4620; 0.9432]
	Life satisfaction (3)	−0.2718	0.7620	[0.4189; 1.3863]
	Life satisfaction (4)	−0.6988	0.4972	[0.3083; 0.8018]
	Life satisfaction (5)	−1.2365	0.2904	[0.1862; 0.4529]
	Life satisfaction (6)	−1.7619	0.1717	[0.1054; 0.2797]
	Life satisfaction (7)	−1.8705	0.1540	[0.0950; 0.2497]

Dispersion effects	Covariate	$\hat{\alpha }$	$\exp\left(\hat{\alpha }\right)$	95% CI
	BMI	−0.0150	0.9851	[0.9619; 1.0088]
	Education	−0.0089	0.9911	[0.9649; 1.0180]
	ADL index	0.0748	1.0776	[0.7289; 1.5932]
	IADL index	0.0188	1.0190	[0.8548; 1.2147]
	Hand grip	0.0123	1.0124	[0.9987; 1.0263]
	Sex (female)	0.3987	1.4899	[1.1069; 2.0054]
	Life satisfaction (2)	0.4022	1.4951	[0.9311; 2.4008]
	Life satisfaction (3)	0.8114	2.2511	[1.3127; 3.8601]
	Life satisfaction (4)	0.6121	1.8442	[1.1486; 2.9611]
	Life satisfaction (5)	0.7163	2.0468	[1.2940; 3.2376]
	Life satisfaction (6)	0.7099	2.0338	[1.2496; 3.3101]
	Life satisfaction (7)	0.3150	1.3703	[0.8585; 2.1873]

*Note:* The model excluded the second level of life satisfaction from the location term and age from the shifting term, resulting in 31 parameters. Parameter estimates were obtained from the training sample; the corresponding standard errors were obtained from the test sample.

As the outcome is measured on an ordinal scale from 1 (excellent) to 5 (poor), higher values indicate worse perception of the individual's health status. Starting with the location‐scale model (Table 4), each additional year of age slightly increases the odds of reporting a poorer health outcome (OR = 1.011, 95% CI: 0.998–1.024). In addition, BMI is positively associated with worse perceived health, with each additional unit increasing the odds by about 3% (OR = 1.032, 95% CI: 1.011–1.053). Education exhibits a protective effect, suggesting that higher educational attainment is associated with lower odds of reporting poorer health. Functional limitations reflected in a higher Activities of Daily Living (ADL) index increase the odds of reporting poorer health (OR = 1.149, 95% CI: 0.960–1.376); the Instrumental of Daily Living (IADL) index is strongly and evidently associated with poorer health, increasing the odds by approximately 28% (OR = 1.283, 95% CI: 1.151–1.430). Physical strength, as measured by hand grip, has a protective effect, with stronger grip reducing the odds of reporting poorer health by about 1.4% per unit (OR = 0.986, 95% CI: 0.974–0.998). Sex differences are also evident, as women exhibit lower odds of reporting poorer health compared to men (OR = 0.752, 95% CI: 0.593–0.954).

Life satisfaction shows a clear monotonic pattern: higher life satisfaction is consistently associated with lower odds of reporting poorer health, with the effect becoming stronger at higher satisfaction levels, from OR = 0.512 (95% CI: 0.312–0.840) at Level 4 to OR = 0.289 (95% CI: 0.142–0.590) at Level 7.

Regarding dispersion effects in the location‐scale model (lower panel of Table 4), most covariates show weak associations with variability. The IADL index slightly increases dispersion (OR = 1.059, 95% CI: 0.989–1.137), while hand grip has no meaningful effect (OR = 0.996, 95% CI: 0.988–1.004). Being female is associated with lower dispersion (OR = 0.847, 95% CI: 0.714–1.005). Higher life satisfaction generally reduces variability, with a relevant reduction observed at Level 5 (OR = 0.692, 95% CI: 0.495–0.969).

Turning to the location‐shift model (Table 5), the effects in the location term are stronger. Age shows evidence for an effect, with each additional year increasing the odds of poorer health by about 2.3% (OR = 1.023, 95% CI: 1.009–1.038). BMI shows a larger detrimental impact than in the location‐scale model (OR = 1.062, 95% CI: 1.035–1.091), while education displays a clearer protective role (OR = 0.966, 95% CI: 0.938–0.995). Functional limitations again play a key role: the ADL index has a positive effect (OR = 1.288, 95% CI: 0.773–2.146), as well as the IADL index, which substantially increases the odds of poorer health (OR = 1.506, 95% CI: 1.200–1.888). Physical strength remains protective (OR = 0.977, 95% CI: 0.961–0.993), and sex differences are more pronounced, with women exhibiting markedly lower odds of reporting poor health (OR = 0.660, 95% CI: 0.462–0.943).

Life satisfaction shows very strong effects in the location‐shift model: from Level 4 onward, the odds of poorer health decrease sharply, from OR = 0.497 (95% CI: 0.308–0.802) at Level 4 to OR = 0.154 (95% CI: 0.095–0.250) at Level 7. In contrast to the location‐scale specification, the dispersion term of the location‐shift model reveals substantial heterogeneity effects. Being female is strongly associated with weaker dispersion among ratings (OR = 1.490, 95% CI: 1.107–2.005). Life satisfaction is also evidently related to decreased variability: intermediate and high satisfaction levels show dispersion odds ratios ranging from OR = 1.844 (95% CI: 1.149–2.961) at Level 4 to OR = 2.251 (95% CI: 1.313–3.860) at Level 3. At the highest satisfaction level, dispersion increases again (OR = 1.370, 95% CI: 0.859–2.187) at Level 7. Overall, explicitly reporting odds ratios and confidence intervals suggests that the location‐shift model captures stronger effects on both the average level and the variability of perceived health than the location‐scale specification.

The variance–covariance matrices of the random intercepts were estimated to be

$$\begin{array}{c} {\hat{\sum }}_{b}=\left(\begin{array}{cc} +\;0.2824 & -0.0092\\ -\;0.0092 & +0.0028 \end{array}\right)\;\left(\text{location}‐\text{scale}\right),\\ {\hat{\sum }}_{b}=\left(\begin{array}{cc} 0.3069 & 0.0582\\ 0.0582 & 0.0370 \end{array}\right)\;\left(\text{location}‐\text{shift}\right). \end{array}$$

They highlight important differences between the two model specifications. In the location‐scale model, heterogeneity across countries mainly affects the location component, while dispersion effects are relatively small and weakly correlated with the average level of perceived health. In contrast, the location‐shift model reveals a larger variability in the dispersion component and a positive association between country‐specific location and dispersion effects. This indicates that countries with worse average self‐reported health also tend to exhibit weaker dispersion in response behavior.

These differences are clearly illustrated in Figure 6 depicting the empirical Bayes estimates of the random intercepts. It presents the location and dispersion effects across different countries, offering insights into how self‐reported health status varies geographically. Countries such as Sweden, Denmark, and Slovakia show both favorable average health assessments and relatively high dispersion, while others, including Latvia, Estonia, and Poland, combine poorer perceived health with more homogeneous response patterns. Overall, Figure 6 highlights that countries differ not only in their average level of self‐reported health but also in how consistently individuals use the response scale, a feature that is more pronounced in the location‐shift specification.

### 4.3 Sensitivity Analysis

To further investigate the findings obtained from the penalized location‐scale and location‐shift models on the SHARE data, we performed two sensitivity analyses with different training‐validation‐test splits. In sensitivity analysis A, we left out all individuals of three countries (Belgium, Czech Republic, and Poland) for testing. In sensitivity analysis B, we left out all individuals of four countries (Italy, Slovenia, Sweden, and Switzerland). In both cases this resulted in a training and validation sample of size $\tilde{n}=1393$ each, and a test sample of $\tilde{n}=643$. Both analyses confirm that only minimal penalization is needed with regard to location effects, but stronger penalization and therefore selection of relevant covariates is deemed necessary with regard to dispersion effects.

#### 4.3.1 Sensitivity Analysis A

According to the predictive log‐likelihood values, the optimal model was obtained for $\lambda_{l}=\exp\left(-10\right),\lambda_{d}=\exp\left(-9\right)$ (location‐shift) and $\lambda_{l}=\exp\left(-8\right),\lambda_{d}=\exp\left(-8\right)$ (location‐scale). As in the main analysis, in both models none of the covariates was excluded from the location term and none of the covariates was excluded from the dispersion term in the location‐shift model, however, the location‐scale model selected only age, IADL index and life satisfaction having dispersion effects. This resulted in an even more parsimonious model specification in the dispersion term with only seven parameters. The estimated variance–covariance matrices of the country‐specific random effects were

$$\begin{array}{cc} {\hat{\sum }}_{b} & =\left(\begin{array}{cc} 0.2745 & 0.0751\\ 0.0751 & 0.0599 \end{array}\right)\;\left(\text{location}‐\text{shift}\right),\\ \;{\hat{\sum }}_{b} & =\left(\begin{array}{cc} +\;0.1891 & -0.0359\\ -\;0.0359 & +0.0126 \end{array}\right)\;\left(\text{location}‐\text{scale}\right), \end{array}$$

which is fully in line with the results of the main analysis. The empirical Bayes estimates, among others, confirmed the positive location effect for Poland ${\hat{b}}_{\text{Poland},\ell }=0.330$ (location‐shift) and ${\hat{b}}_{\text{Poland},\ell }=0.376$ (location‐scale) indicating poorer perceived health. On the test sample including data from Belgium, Czech Republic and Poland, the average RPS was 0.4376 (location‐shift) and 0.4385 (location‐scale), which indicates comparable predictive accuracy as in the main analysis.

#### 4.3.2 Sensitivity Analysis B

According to the predictive log‐likelihood values, the optimal model was obtained for $\lambda_{l}=\exp\left(-7\right),\lambda_{d}=\exp\left(-7\right)$ (location‐shift) and $\lambda_{l}=\exp\left(-10\right),\lambda_{d}=\exp\left(-7\right)$ (location‐scale), which again did not yield variable selection in the location term, but a fusion of levels 9 and 10 (location‐shift) and levels 8 and 9 (location‐scale) of life satisfaction. Regarding the dispersion term, only ADL index was excluded in the location‐shift model, while in the location‐scale model ADL index, BMI and hand grip were excluded having no dispersion effect. The estimated variance–covariance matrices of the country‐specific random effects were

$$\begin{array}{cc} {\hat{\sum }}_{b} & =\left(\begin{array}{cc} 0.3866 & 0.0757\\ 0.0757 & 0.0943 \end{array}\right)\;\left(\text{location}‐\text{shift}\right),\\ \;{\hat{\sum }}_{b} & =\left(\begin{array}{cc} +\;0.0511 & -0.0158\\ -\;0.0158 & +0.0111 \end{array}\right)\;\left(\text{location}‐\text{scale}\right), \end{array}$$

which supports the apparent variability in the location term according to the location‐shift model. The empirical Bayes estimates, among others, confirmed the strong, negative location effect for Switzerland ${\hat{b}}_{\text{Switzerland},\ell }=-1.329$ (location‐shift) and ${\hat{b}}_{\text{Switzerland},\ell }=-0.135$ (location‐scale) indicating much better perceived health compared to other countries (cf. also Figure 2). On the test sample including data from Italy, Slovenia, Sweden, Switzerland, the average RPS was 0.4915 (location‐shift) and 0.4882 (location‐scale), which is negligibly smaller than in the main analysis and underlines the closeness of the two models in terms of prediction.

Overall, the two sensitivity analyses showed a consistent picture together with the main analysis and demonstrate the good performance regarding out‐of‐sample prediction. Regarding the dispersion term, the location‐scale model yielded more parsimonious solutions throughout.

## 5 Simulation Study

To underpin the results of the case study, we conducted two simulation studies to investigate the performance of the proposed penalized mixed‐effects models. Our aim was to analyze the model fit and to evaluate the variable selection mechanism by the adaptive penalty. In the first simulation scenario, the data was generated from the mixed‐effects location‐shift model, and in the second scenario, the data was generated from the mixed‐effects location‐scale model.

### 5.1 Experimental Design

We simulated clustered data with response variable $y_{\mathrm{ij}}\in \left\{1,2,3\right\},i=1,\ldots ,n,j=1,\ldots ,N_{i}$. In *setting 1* we set $n=50,N_{i}=25$ and in *setting 2* we set $n=30,N_{i}=40$, resulting in total sample sizes comparable to our case study. We considered two uniformly distributed covariates $x_{\mathrm{ij}1},x_{\mathrm{ij}2}∼U\left[-1,1\right]$ and one ordinal covariate $x_{\mathrm{ij}3}\in \left\{1,2,3,4\right\}$ with the first level serving as the reference category. Further, we assumed that $x_{\mathrm{ij}}=z_{\mathrm{ij}}$. Random intercepts were drawn from a normal distribution with mean 0 and variance–covariance matrix

$$\sum_{b}=\left(\begin{array}{cc} 0.10 & 0.02\\ 0.02 & 0.10 \end{array}\right).$$

The intercepts were set to $\beta_{01}=-1,\beta_{02}=1$, and the regression coefficients were set to $\beta ={\left(-0.2,0.2,-0.5,0.5,0.5\right)}^{⊤}$, $\alpha ={\left(-0.4,0.4,0,0,0\right)}^{⊤}$. Hence, regarding $x_{3}$ the third and fourth level had the same location effect and there was no dispersion effect. In each simulation run (100 replications) we draw a training sample for fitting and a validation sample of the same size to determine the optimal tuning parameters $\lambda_{l}$ and $\lambda_{d}$. For this we optimized the predictive log‐likelihood values on a two‐dimensional grid with values $\lambda_{l},\lambda_{d}\in \left\{-10,-2\right\}$ (on the logarithmic scale).

For covariates $x_{1}$ and $x_{2}$ we calculated the true positive rate (TPR) and the false negative rate (FNR), as the proportions over 100 replications, in the location term as well as in the dispersion term. For covariate $x_{3}$ we calculated the TPR and the FNR in the location term, as well as the true negative rate (TNR) and false positive rate (FPR) in the dispersion term. We also evaluated how often $\beta_{33}$ and $\beta_{34}$ were correctly collapsed to one category.

### 5.2 Results

**FIGURE 7:**
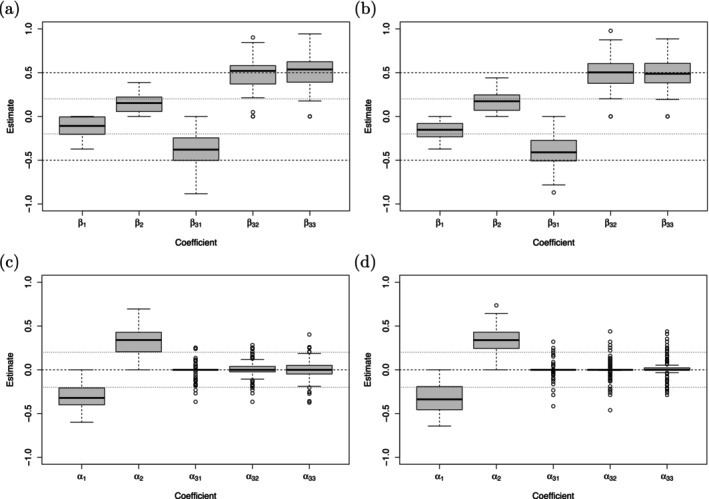
Simulation study. Coefficient estimates of the location effects (upper panels) and dispersion effects (lower panels) for the mixed‐effects location‐shift model based on 100 replications. Panels (a) and (c) refer to the setting with $n=50,N_{i}=25$, panels (b) and (d) refer to the setting with $n=30,N_{i}=40$. The true coefficients are marked by dashed and dotted lines, respectively.

**FIGURE 8:**
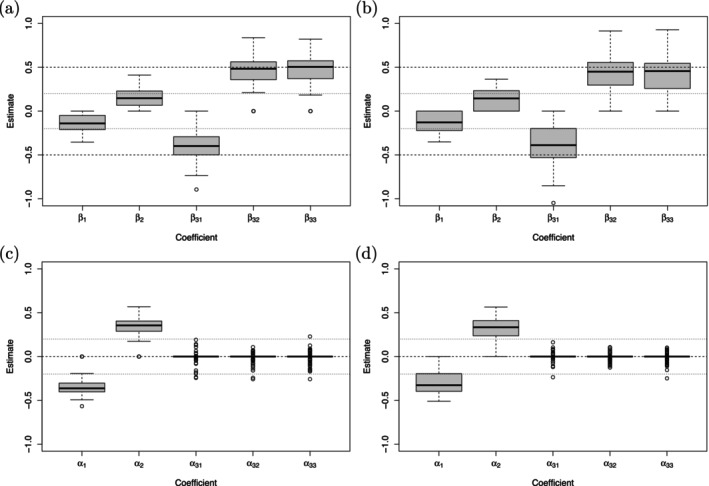
Simulation study. Coefficient estimates of the location effects (upper panels) and dispersion effects (lower panels) for the mixed‐effects location‐scale model based on 100 replications. Panels (a) and (c) refer to the setting with $n=50,N_{i}=25$, panels (b) and (d) refer to the setting with $n=30,N_{i}=40$. The true coefficients are marked by dashed and dotted lines, respectively.

The coefficient estimates for the location‐shift model shown in Figure 7 and for the location‐scale model shown in Figure 8 indicate that the fitting procedure performed very well. As expected, because of the penalized fit, the coefficient estimates of the non‐zero coefficients were downward (location effects) or upward (dispersion effects) biased. The boxplots reveal that the exclusion of the dispersion effect in the ordinal covariate $x_{3}$ worked better in the location‐scale model (see lower panels in Figure 8). This is also seen from the variable selection rates depicted in Tables 6 and 7 with TNR for $x_{3}$ between 0.66 and 0.81. It supports the findings in the SHARE study with a sparser representation of the dispersion term in the location‐scale model. The proportion of simulation runs in which $\beta_{33}$ and $\beta_{34}$ were correctly collapsed to one category varied between 0.52 and 0.63, which proved to be challenging. The TPR for the non‐zero coefficients varied between 0.76 and 0.91 (location‐shift) and between 0.70 and 0.87 (location‐scale) underlying an overall sufficient performance.

**TABLE 6:** Simulation study. Variable selection rates for of the location effects and dispersion effects for the mixed‐effects location‐shift model based on 100 replications.

Setting	Covariate	Location		Dispersion	
		TPR	FNR	TPR	FNR
(1) $n=50,N_{i}=25$	$x_{1}$	0.76	0.24	0.89	0.11
	$x_{2}$	0.82	0.18	0.86	0.14
(2) $n=30,N_{i}=40$	$x_{1}$	0.81	0.19	0.86	0.14
	$x_{2}$	0.80	0.20	0.89	0.11

		TPR	FNR	TNR	FPR
(1) $n=50,N_{i}=25$	$x_{3}$	0.89	0.11	0.66	0.34
(2) $n=30,N_{i}=40$	$x_{3}$	0.91	0.09	0.74	0.26

*Note:* The true regression coefficients were $\beta ={\left(-0.2,0.2,-0.5,0.5,0.5\right)}^{⊤}$, $\alpha ={\left(-0.4,0.4,0,0,0\right)}^{⊤}$.

**TABLE 7:** Simulation study. Variable selection rates for of the location effects and dispersion effects for the mixed‐effects location‐scale model based on 100 replications.

Setting	Covariate	Location		Dispersion	
		TPR	FNR	TPR	FNR
(1) $n=50,N_{i}=25$	$x_{1}$	0.78	0.22	0.87	0.13
	$x_{2}$	0.82	0.18	0.87	0.13
(2) $n=30,N_{i}=40$	$x_{1}$	0.70	0.30	0.80	0.20
	$x_{2}$	0.75	0.25	0.80	0.20

		TPR	FNR	TNR	FPR
(1) $n=50,N_{i}=25$	$x_{3}$	0.87	0.13	0.77	0.23
(2) $n=30,N_{i}=40$	$x_{3}$	0.80	0.20	0.81	0.19

*Note:* The true regression coefficients were $\beta ={\left(-0.2,0.2,-0.5,0.5,0.5\right)}^{⊤}$, $\alpha ={\left(-0.4,0.4,0,0,0\right)}^{⊤}$.

## 6 Conclusion

This paper introduces advanced cumulative regression models designed for the analysis of ordinal outcomes when data come in clusters of observation units. We present an in‐depth case study on self‐reported health assessments across European countries using data from the SHARE study. The penalized location‐scale and location‐shift models with country‐specific intercepts reveal several important findings on the effect of age, sex, BMI, education, physical strength and functional limitations, as well as geographical differences. Taking together the findings of the main analysis and the two sensitivity analyses, there is apparent evidence for location and dispersion effects of the IADL index, sex and life satisfaction, as well as a location effect of BMI. By accounting for both location effects (systematic shifts in central tendency) and dispersion effects (response variability across individuals), the model provides a more comprehensive understanding of health perceptions. This underlines that classical cumulative models, which assume homogeneous dispersion, may fail to capture important heterogeneity in self‐perceived health status (factors that drive central tendencies and response variability), leading to biased or incomplete conclusions. In comparing the two approaches, we also stress that when differences in global fit indices are negligible, the choice of model should be driven mainly by the research objectives and the interpretability of the results, rather than by marginal gains in information criteria.

A key methodological contribution of this work is the use of adaptive penalized likelihood estimation to perform variable selection in both the location and dispersion components. By applying LASSO‐type penalties ($L_{1}$ and fusion penalty terms), the model automatically identifies the most relevant covariates while ensuring parsimony and avoiding overfitting. The sufficient performance of the adaptive penalties were empirically confirmed in our simulation study. The approach is highly relevant in the considered models, because already with a moderate number of covariates the location‐scale and location‐shift models may result in a large number of parameters to be estimated compared to the sample size. Although the proposed models are not restricted to linear predictor functions, in the SHARE application we limited our analysis to linear effects for continuous covariates. Models including dispersion effects already involve 33 parameters, and this number would increase substantially when incorporating polynomial terms or spline functions. Such complexity would considerably increase computational time and would require an effect selection procedure, which is not yet available in the current implementation.

A decisive part of the penalized fitting procedure is the choice of the optimal tuning parameter. In our evaluations we used a simple training‐validation‐test split and computed the predictive log‐likelihood. Improved, more demanding procedures, like leave‐one‐out cross‐validation, repeated cross‐validation, or bootstrapping, could be investigated prospectively, but are limited because of the already very high level of computational complexity for fitting the proposed models. Computational complexity and the runtime by our implementation of SAS PROC NLMIXED was also the reason why we presented only two data splits as sensitivity analyses in the SHARE study, but not a more complex resampling experiment.

Future research should explore extensions of this framework to longitudinal data, investigating how both location and dispersion effects evolve over time. Additionally, incorporating alternative penalty structures (like grouped penalties, see [35, 45]) could further refine variable selection techniques in mixed location‐scale and location‐shift models. Finally, the integration of Bayesian regularization methods or nonparametric approaches (see the tree‐structured location‐scale model by [46]) may provide new insights into the uncertainty quantification of model parameters. By advancing these methodological directions, future studies can further improve statistical tools for analyzing ordinal data with complex dependency structures.

## Funding

This work was supported by Deutsche Forschungsgemeinschaft (BE 7543/1).

## Conflicts of Interest

The authors declare no conflicts of interest.

## Data Availability

The data that support the findings of this study are openly available in SHARE Research Data Center at https://share‐eric.eu/data/data‐access/conditions‐of‐use.

## ENDNOTES

^1^

https://share‐eric.eu/.

## Appendix A Implementation by SAS PROC NLMIXED

### Random Effects Location‐Shift Model

%macro locshiftFLASSO(dataset, dispersion, maxiter, maxfunc, lambda_l, lambda_d, gamma10, gamma20, gamma30, gamma40,

b_age0, b_bmi0, b_gender0, b_yedu0, b_adl0, b_iadl0, b_maxgrip0, b_ls50, b_ls65, b_ls76, b_ls87, b_ls98, b_ls109,

b_ls60, b_ls70, b_ls80, b_ls90, b_ls100, d_age0, d_bmi0, d_gender0, d_yedu0, d_adl0, d_iadl0, d_maxgrip0, d_ls50,

d_ls65, d_ls76, d_ls87, d_ls98, d_ls109, d_ls60, d_ls70, d_ls80, d_ls90, d_ls100, s_loc0, s_disp0, cov0);

PROC NLMIXED data=&dataset GCONV=1e‐12 FCONV=1e‐12 MAXITER=&maxiter MAXFUNC=&maxfunc tech=quanew method=gauss qpoints=1;

PARMS gamma1=&gamma10 gamma2=&gamma20 gamma3=&gamma30 gamma4=&gamma40

b_age=&b_age0 b_bmi=&b_bmi0 b_gender=&b_gender0 b_yedu=&b_yedu0 b_adl=&b_adl0 b_iadl=&b_iadl0 b_maxgrip=&b_maxgrip0

b_ls5=&b_ls50 b_ls6=&b_ls60 b_ls7=&b_ls70 b_ls8=&b_ls80 b_ls9=&b_ls90 b_ls10=&b_ls100 s_loc=&s_loc0

d_age=&d_age0 d_bmi=&d_bmi0 d_gender=&d_gender0 d_yedu=&d_yedu0 d_adl=&d_adl0 d_iadl=&d_iadl0 d_maxgrip=&d_maxgrip0

d_ls5=&d_ls50 d_ls6=&d_ls60 d_ls7=&d_ls70 d_ls8=&d_ls80 d_ls9=&d_ls90 d_ls10=&d_ls100 s_disp=&s_disp0 cov=&cov0;

mean = age*b_age+bmi*b_bmi+gender*b_gender+yedu*b_yedu+adl*b_adl+iadl*b_iadl+maxgrip*b_maxgrip

+lifesat5*b_ls5+lifesat6*b_ls6+lifesat7*b_ls7+lifesat8*b_ls8+lifesat9*b_ls9+lifesat10*b_ls10+b0;

dispersion=&dispersion;

IF dispersion=1 THEN disp = age*d_age+bmi*d_bmi+gender*d_gender+yedu*d_yedu+adl*d_adl+iadl*d_iadl+maxgrip*d_maxgrip

+lifesat5*d_ls5+lifesat6*d_ls6+lifesat7*d_ls7+lifesat8*d_ls8+lifesat9*d_ls9+lifesat10*d_ls10+w0;

ELSE IF dispersion=0 THEN disp = 0;

clogit1 = gamma1 ‐ mean ‐ 1.5*disp;

clogit2 = gamma2 ‐ mean ‐ 0.5*disp;

clogit3 = gamma3 ‐ mean + 0.5*disp;

clogit4 = gamma4 ‐ mean + 1.5*disp;

cprob1 = 1/(1 + EXP(‐clogit1));

cprob2 = 1/(1 + EXP(‐clogit2));

cprob3 = 1/(1 + EXP(‐clogit3));

cprob4 = 1/(1 + EXP(‐clogit4));

IF (sphus=1) THEN p = cprob1;

ELSE IF (sphus=2) THEN p = cprob2 ‐ cprob1;

ELSE IF (sphus=3) THEN p = cprob3 ‐ cprob2;

ELSE IF (sphus=4) THEN p = cprob4 ‐ cprob3;

ELSE IF (sphus=5) THEN p = 1 ‐ cprob4;

pen_l=&lambda_l;

pen_d=&lambda_d;

b_age0=&b_age0; b_bmi0=&b_bmi0; b_gender0=&b_gender0; b_yedu0=&b_yedu0; b_adl0=&b_adl0; b_iadl0=&b_iadl0;

b_maxgrip0=&b_maxgrip0; b_ls50=&b_ls50; b_ls65=&b_ls65; b_ls76=&b_ls76; b_ls87=&b_ls87; b_ls98=&b_ls98; b_ls109=&b_ls109;

d_age0=&d_age0; d_bmi0=&d_bmi0; d_gender0=&d_gender0; d_yedu0=&d_yedu0; d_adl0=&d_adl0; d_iadl0=&d_iadl0;

d_maxgrip0=&d_maxgrip0; d_ls50=&d_ls50; d_ls65=&d_ls65; d_ls76=&d_ls76; d_ls87=&d_ls87; d_ls98=&d_ls98; d_ls109=&d_ls109;

IF (pen_l=0 AND pen_d=0) THEN logl = LOG(p);

ELSE IF (dispersion=1 AND pen_d>0) THEN logl = LOG(p) ‐ pen_l*sumabs(1/abs(b_ls50)*b_ls5, 1/abs(b_ls65)*(b_ls6‐

b_ls5),

1/abs(b_ls76)*(b_ls7‐b_ls6), 1/abs(b_ls87)*(b_ls8‐b_ls7), 1/abs(b_ls98)*(b_ls9‐b_ls8), 1/abs(b_ls109)*(b_ls10‐b_ls9))

‐ pen_l*sumabs(1/abs(b_age0)*b_age, 1/abs(b_bmi0)*b_bmi, 1/abs(b_gender0)*b_gender, 1/abs(b_yedu0)*b_yedu,

1/abs(b_adl0)*b_adl, 1/abs(b_iadl0)*b_iadl, 1/abs(b_maxgrip0)*b_maxgrip)

‐ pen_d*sumabs(1/abs(d_ls50)*d_ls5, 1/abs(d_ls65)*(d_ls6‐d_ls5), 1/abs(d_ls76)*(d_ls7‐d_ls6), 1/abs(d_ls87)*(d_ls8‐

d_ls7),

1/abs(d_ls98)*(d_ls9‐d_ls8), 1/abs(d_ls109)*(d_ls10‐d_ls9))

‐ pen_d*sumabs(1/abs(d_age0)*d_age, 1/abs(d_bmi0)*d_bmi, 1/abs(d_gender0)*d_gender, 1/abs(d_yedu0)*d_yedu,

1/abs(d_adl0)*d_adl, 1/abs(d_iadl0)*d_iadl, 1/abs(d_maxgrip0)*d_maxgrip);

ELSE IF (dispersion=0 OR pen_d=0) THEN logl = LOG(p) ‐ pen_l*sumabs(1/abs(b_ls50)*b_ls5, 1/abs(b_ls65)*(b_ls6‐b_ls5),

1/abs(b_ls76)*(b_ls7‐b_ls6), 1/abs(b_ls87)*(b_ls8‐b_ls7), 1/abs(b_ls98)*(b_ls9‐b_ls8), 1/abs(b_ls109)*(b_ls10‐b_ls9))

‐ pen_l*sumabs(1/abs(b_age0)*b_age, 1/abs(b_bmi0)*b_bmi, 1/abs(b_gender0)*b_gender, 1/abs(b_yedu0)*b_yedu,

1/abs(b_adl0)*b_adl, 1/abs(b_iadl0)*b_iadl, 1/abs(b_maxgrip0)*b_maxgrip);

MODEL sphus ∼ GENERAL(logl);

RANDOM b0 w0 ∼ NORMAL([0,0], [s_loc, cov, s_disp])

 
SUBJECT=country;

 
ods output parameterestimates=param_shift;

 
ods output FitStatistics=fit_shift;

RUN;

%mend;

### Random Effects Location‐Scale Model

%macro locscaleFLASSO(dataset, dispersion, maxiter, maxfunc, lambda_l, lambda_d, gamma10, gamma20, gamma30, gamma40,

b_age0, b_bmi0, b_gender0, b_yedu0, b_adl0, b_iadl0, b_maxgrip0, b_ls50, b_ls65, b_ls76, b_ls87, b_ls98, b_ls109,

b_ls60, b_ls70, b_ls80, b_ls90, b_ls100, d_age0, d_bmi0, d_gender0, d_yedu0, d_adl0, d_iadl0, d_maxgrip0, d_ls50,

d_ls65, d_ls76, d_ls87, d_ls98, d_ls109, d_ls60, d_ls70, d_ls80, d_ls90, d_ls100, s_loc0, s_disp0, cov0);

PROC NLMIXED data=&dataset GCONV=1e‐12 FCONV=1e‐12 MAXITER=&maxiter MAXFUNC=&maxfunc tech=quanew method=gauss qpoints=1;

PARMS gamma1=&gamma10 gamma2=&gamma20 gamma3=&gamma30 gamma4=&gamma40

b_age=&b_age0 b_bmi=&b_bmi0 b_gender=&b_gender0 b_yedu=&b_yedu0 b_adl=&b_adl0 b_iadl=&b_iadl0 b_maxgrip=&b_maxgrip0

b_ls5=&b_ls50 b_ls6=&b_ls60 b_ls7=&b_ls70 b_ls8=&b_ls80 b_ls9=&b_ls90 b_ls10=&b_ls100 s_loc=&s_loc0

d_age=&d_age0 d_bmi=&d_bmi0 d_gender=&d_gender0 d_yedu=&d_yedu0 d_adl=&d_adl0 d_iadl=&d_iadl0 d_maxgrip=&d_maxgrip0

d_ls5=&d_ls50 d_ls6=&d_ls60 d_ls7=&d_ls70 d_ls8=&d_ls80 d_ls9=&d_ls90 d_ls10=&d_ls100 s_disp=&s_disp0 cov=&cov0;

mean = age*b_age+bmi*b_bmi+gender*b_gender+yedu*b_yedu+adl*b_adl+iadl*b_iadl+maxgrip*b_maxgrip

+lifesat5*b_ls5+lifesat6*b_ls6+lifesat7*b_ls7+lifesat8*b_ls8+lifesat9*b_ls9+lifesat10*b_ls10+b0;

dispersion=&dispersion;

IF dispersion=1 THEN disp = EXP(age*d_age+bmi*d_bmi+gender*d_gender+yedu*d_yedu+adl*d_adl+iadl*d_iadl+maxgrip*d_maxgrip

+lifesat5*d_ls5+lifesat6*d_ls6+lifesat7*d_ls7+lifesat8*d_ls8+lifesat9*d_ls9+lifesat10*d_ls10+w0);

ELSE IF dispersion=0 THEN disp = EXP(0);

clogit1 = (gamma1 ‐ mean)/disp;

clogit2 = (gamma2 ‐ mean)/disp;

clogit3 = (gamma3 ‐ mean)/disp;

clogit4 = (gamma4 ‐ mean)/disp;

cprob1 = 1/(1 + EXP(‐clogit1));

cprob2 = 1/(1 + EXP(‐clogit2));

cprob3 = 1/(1 + EXP(‐clogit3));

cprob4 = 1/(1 + EXP(‐clogit4));

IF (sphus=1) THEN p = cprob1;

ELSE IF (sphus=2) THEN p = cprob2 ‐ cprob1;

ELSE IF (sphus=3) THEN p = cprob3 ‐ cprob2;

ELSE IF (sphus=4) THEN p = cprob4 ‐ cprob3;

ELSE IF (sphus=5) THEN p = 1 ‐ cprob4;

pen_l=&lambda_l;

pen_d=&lambda_d;

b_age0=&b_age0; b_bmi0=&b_bmi0; b_gender0=&b_gender0; b_yedu0=&b_yedu0; b_adl0=&b_adl0; b_iadl0=&b_iadl0;

b_maxgrip0=&b_maxgrip0; b_ls50=&b_ls50; b_ls65=&b_ls65; b_ls76=&b_ls76; b_ls87=&b_ls87; b_ls98=&b_ls98; b_ls109=&b_ls109;

d_age0=&d_age0; d_bmi0=&d_bmi0; d_gender0=&d_gender0; d_yedu0=&d_yedu0; d_adl0=&d_adl0; d_iadl0=&d_iadl0;

d_maxgrip0=&d_maxgrip0; d_ls50=&d_ls50; d_ls65=&d_ls65; d_ls76=&d_ls76; d_ls87=&d_ls87; d_ls98=&d_ls98; d_ls109=&d_ls109;

IF (pen_l=0 AND pen_d=0) THEN logl = LOG(p);

ELSE IF (dispersion=1 AND pen_d>0) THEN logl = LOG(p) ‐ pen_l*sumabs(1/abs(b_ls50)*b_ls5, 1/abs(b_ls65)*(b_ls6‐

b_ls5),

1/abs(b_ls76)*(b_ls7‐b_ls6), 1/abs(b_ls87)*(b_ls8‐b_ls7), 1/abs(b_ls98)*(b_ls9‐b_ls8), 1/abs(b_ls109)*(b_ls10‐b_ls9))

‐ pen_l*sumabs(1/abs(b_age0)*b_age, 1/abs(b_bmi0)*b_bmi, 1/abs(b_gender0)*b_gender, 1/abs(b_yedu0)*b_yedu,

1/abs(b_adl0)*b_adl, 1/abs(b_iadl0)*b_iadl, 1/abs(b_maxgrip0)*b_maxgrip)

‐ pen_d*sumabs(1/abs(d_ls50)*d_ls5, 1/abs(d_ls65)*(d_ls6‐d_ls5), 1/abs(d_ls76)*(d_ls7‐d_ls6), 1/abs(d_ls87)*(d_ls8‐

d_ls7),

1/abs(d_ls98)*(d_ls9‐d_ls8), 1/abs(d_ls109)*(d_ls10‐d_ls9))

‐ pen_d*sumabs(1/abs(d_age0)*d_age, 1/abs(d_bmi0)*d_bmi, 1/abs(d_gender0)*d_gender, 1/abs(d_yedu0)*d_yedu,

1/abs(d_adl0)*d_adl, 1/abs(d_iadl0)*d_iadl, 1/abs(d_maxgrip0)*d_maxgrip);

ELSE IF (dispersion=0 OR pen_d=0) THEN logl = LOG(p) ‐ pen_l*sumabs(1/abs(b_ls50)*b_ls5, 1/abs(b_ls65)*(b_ls6‐b_ls5),

1/abs(b_ls76)*(b_ls7‐b_ls6), 1/abs(b_ls87)*(b_ls8‐b_ls7), 1/abs(b_ls98)*(b_ls9‐b_ls8), 1/abs(b_ls109)*(b_ls10‐b_ls9))

‐ pen_l*sumabs(1/abs(b_age0)*b_age, 1/abs(b_bmi0)*b_bmi, 1/abs(b_gender0)*b_gender, 1/abs(b_yedu0)*b_yedu,

1/abs(b_adl0)*b_adl, 1/abs(b_iadl0)*b_iadl, 1/abs(b_maxgrip0)*b_maxgrip);

MODEL sphus ∼ GENERAL(logl);

RANDOM b0 w0 ∼ NORMAL([0,0], [s_loc, cov, s_disp])

 
SUBJECT=country;

 
ods output parameterestimates=param_scale;

 
ods output FitStatistics=fit_scale;

RUN;

%mend;
